# Impact of carbonic anhydrase 9 gene polymorphism on the progression of colorectal cancer

**DOI:** 10.7150/jca.73898

**Published:** 2022-06-21

**Authors:** Hsien-Cheng Huang, Bei-Hao Shiu, Yasser Nassef, Chi-Chou Huang, Ying-Erh Chou, Wen-Chien Ting, Lun-Ching Chang, Jian-Cheng Lin, Li-Kai Hsiao, Shun-Fa Yang, Shih-Chi Su

**Affiliations:** 1 Institute of Medicine, Chung Shan Medical University, Taichung, Taiwan.; 2 Department of Emergency Medicine, Kuang Tien General Hospital, Taichung, Taiwan.; 3 Department of Surgery, Chung Shan Medical University Hospital, Taichung, Taiwan.; 4 School of Medicine, Chung Shan Medical University, Taichung, Taiwan.; 5 Department of Mathematical Sciences, Florida Atlantic University, Florida, USA.; 6 Department of Medical Research, Chung Shan Medical University Hospital, Taichung, Taiwan.; 7 MingDao High School, Taichung, Taiwan.; 8 Whole-Genome Research Core Laboratory of Human Diseases, Chang Gung Memorial Hospital, Keelung, Taiwan.; 9 Department of Dermatology, Drug Hypersensitivity Clinical and Research Center, Chang Gung Memorial Hospital, Linkou, Taiwan.

**Keywords:** Colorectal cancer, carbonic anhydrase 9, single-nucleotide polymorphism, tumor differentiation

## Abstract

Colorectal cancer (CRC) is a commonly occurring tumor type worldwide, and its development is governed by a connection between genetic variations and acquired factors. Carbonic anhydrase 9 (CA9) is a cell-surface pH modulator that has been demonstrated to contribute to key steps of cancer progression. Here, we attempted to interrogate the effect of *CA9* gene polymorphisms on the development of CRC in 470 cases and 470 gender- and age-matched non-cancer controls. We found that none of three *CA9* single-nucleotide polymorphisms (SNPs) tested, including rs2071676, rs3829078, and rs1048638, was significantly associated with the occurrence of CRC. Yet, while evaluating the clinicopathological variables, cases carrying at least one reference allele (G allele) of rs2071676 tended to develop poorly differentiated tumors less frequently than those who are homozygous for the alternative allele (A allele) of rs2071676 (GA+GG vs AA; OR, 0.483; 95% CI, 0.242-0.963; *p*=0.036). Further stratification revealed that as compared to homozygous carriers of the alternative allele (AA), cases of colon cancer bearing at least one reference allele of rs2071676 (GA+GG) less frequently developed poorly differentiated tumors (OR, 0.449; 95% CI, 0.221-0.911; *p*=0.024) and lymphovascular invasion (OR, 0.570; 95% CI, 0.361-0.900; *p*=0.015). Such genetic effect was exclusively observed in colon cancer but not in rectal cancer. Our results indicate an anatomical site-specific impact of *CA9* gene polymorphisms on modulating the progression of colorectal malignancies.

## Introduction

Nowadays, colorectal cancer (CRC) is among the most common neoplasms globally 1, and this burden is expected to reach 3.2 million new cases by 2040 2. In Taiwan, being the most frequent malignancy in men and the second in women, CRC remains a growing national public health challenge, accounting for a significant portion of cancer-related deaths 3. In spite of the recent progresses in therapeutic approaches and cancer etiology, a consistent increase in the age-standardized death rate of colon cancer was observed over the years in Taiwan 4. A plethora of risk parameters were known to contribute to such high prevalence and mortality. Diet and lifestyle choices with excess exposure of tumor-causing agents, such as smoking and alcohol drinking, have long been recognized as key environmental causes of CRC 5. In addition, various inherited alterations that affect angiogenesis, adhesion, proteolysis, and cell growth have been shown to modulate CRC carcinogenesis 6. Aside from host factors, studies of CRC etiology currently lay a greater emphasis on a shift of the gut commensal microbiome, which has been proposed to lie at the intersection of those potential risks mentioned above 7. Given the heterogeneous nature of colorectal tumorigenesis, all these susceptibility factors seem to be interconnected and required to evaluate the disease prognosis.

Hypoxia is a unique feature of cancer niche during multistage carcinogenesis. In the absence of oxygen, cancer cells rely on aerobic glycolysis or the Warburg effect 8 for tumor expansion. Such metabolic reprogramming creates an acidic tumor microenvironment, where cells are needed to enhance the activity of pH-regulating machinery to avoid prolonged intracellular acidosis 9. Carbonic anhydrase 9 (CA9), belonging to the α carbonic anhydrase family of zinc metalloenzymes that catalyze the reversible hydration of carbon dioxide to bicarbonate ions and protons, is a cell-surface glycoprotein that is upregulated by hypoxia and implicated in adaptation to acidosis 10. Aside from acting as a pH modulator, CA9 can also function as an adhesion molecule, mediating the assembly and maturation of focal contacts during cell spreading and migration 11. Converging observations have indicated that CA9 is functionally involved in diverse hallmarks of cancer, such as promotion of primary tumor growth and metastatic dissemination 12. In the prognosis of CRC, high expression of CA9 was found to be associated with a poor outcome 13, 14, suggesting its translational value in CRC management. Recently, a growing number of studies have unveiled the associations of *CA9* gene polymorphisms with the risk, progression, or therapy outcome of various malignancies 15-20. Yet, the influence of *CA9* gene variants on the susceptibility to colon cancer remains mostly unexplored. Here, we conducted a case-control study to interrogate how and to what extent *CA9* single-nucleotide polymorphisms (SNPs) affect the progression of CRC.

## Materials and Methods

### Subjects

A total of 470 patients with CRC and 470 cancer-free controls were recruited in this investigation, with the approval by the institutional review board of Chung Shan Medical University Hospital in Taichung, Taiwan. All subjects, enrolled from 2016 to 2021, provided informed written consent at enrollment. Moreover, subjects with history of cancer of any sites and self-reported diseases such as cardiovascular, diabetes, and autoimmune diseases were excluded from the control group. Clinical staging of CRC was staged determined according to the TNM staging system of the American Joint Committee on Cancer (AJCC) 21 at the time of diagnosis. Cancer differentiation was evaluated by a pathologist and graded according to the AJCC classification. Demographic data on age and gender were recorded from each participant.

### Genotyping

Genomic DNA derived from the whole blood was isolated by using QIAamp DNA blood mini kits (Qiagen, Valencia, CA, USA) 22, 23. Based on the previous research, three genetic variants of CA9 SNPs (rs2071676, rs3829078, and rs1048638) were selected 15-17, 24, 25. Discrimination of polymorphic alleles for three *CA9* SNPs (rs2071676, rs3829078, and rs1048638) was performed through the TaqMan assay with an ABI StepOne™ Real-Time PCR System (Applied Biosystems, Foster City, CA, USA), and further assessed with SDS version 3.0 software (Applied Biosystems).

### Statistical analysis

The Hardy-Weinberg equilibrium for biallelic markers was assessed using a chi-square goodness-of-fit test. The comparison of demographic factors between CRC patients and controls was carried out using Fisher's exact test. The adjusted odds ratios (AORs) with their 95% confidence intervals (CIs) for the genetic association of *CA9* SNPs with and the predisposition to CRC were evaluated by multiple logistic regression models after controlling for age and gender. Data were calculated by using SAS statistical software (Version 9.1, 2005; SAS Institute Inc., Cary, NC). A *p* value < 0.05 was considered significant.

## Results

### Demographic and clinical characteristics of study cohorts

To assess the influence of CA9 gene polymorphisms on the risk and progression of colorectal tumor, 470 patients with CRC were enrolled in this investigation. As age and gender are known to affect the predisposition to CRC 26, 470 cancer-free subjects who matched age and gender to the cases were recruited to exclude possible confounders. The comparison of demographic and clinical features between the case and control group was shown in **Table 1**. Within the case group, 107 and 363 suffered from tumors of the rectum and colon, respectively. 48.4% and 16.7% of cases developed lymphatic spread and distal metastasis, respectively.

### CA9 gene polymorphisms were associated with the progression but not the occurrence of CRC

To explore how and to what extent *CA9* gene variations influence the development of CRC, three *CA9* SNPs (rs2071676, rs3829078, and rs1048638) were chosen according to their broad correlations with the risk of many malignancies 16-18 and genotyped in the present study. The genotype ratios for individual SNP in our cohorts were examined (**Table 2**). No deviation (*p*>0.05) from Hardy-Weinberg equilibrium in both study groups was identified for all three SNPs. We found that none of these *CA9* variants was significantly associated with the occurrence of CRC in our cohorts. Additionally, we tested whether genetic polymorphisms of* CA9* were connected to the clinicopathological features of CRC patients. We found that cases who possess at least one reference allele (G allele) of rs2071676 (GA+GG) tended to develop poorly differentiated tumors less frequently than those who are homozygous for the alternative allele (A allele) of rs2071676 (AA) (OR, 0.483; 95% CI, 0.242-0.963; *p*=0.036) (**Table 3**). Although not statistically significant, homozygotes for the alternative allele of rs2071676 (AA) are marginally associated with advanced cancers, with an inclination to develop large-size tumors and potentiate lymphovascular and perineural invasion. These results indicate a role of *CA9* gene polymorphisms in CRC progression.

### rs2071676 was associated exclusively with tumors of the colon, but not that of the rectum

Since a missense SNP, rs2071676, was noted to be associated with CRC differentiation, we next tested whether this genetic effect was specific to the tumor location. Our stratification analyses revealed that as compared to the homozygous carriers of the alternative allele of rs2071676 (AA), cases of colon cancer carrying at least one reference allele of rs2071676 (GA+GG) less frequently developed poorly differentiated tumors (OR, 0.449; 95% CI, 0.221-0.911; *p*=0.024) and lymphovascular invasion (OR, 0.570; 95% CI, 0.361-0.900; *p*=0.015) (**Table 4**). This genetic association was exclusively observed in colon cancer but not in rectal cancer. Even though there was no statistical significance, a marginally protective effect of rs2071676 G allele on the progression into advanced colon cancer (e.g. large-size tumors or perineural invasion) was seen. Overall, there data unveil a genetic interaction of *CA9* with the tumor differentiation and lymphovascular invasion of colon adenocarcinoma.

## Discussion

The progression of CRC is a multistep process governed by an intricate combination of environmental and genetic factors. Here, we demonstrated that *CA9* gene polymorphisms, rs2071676, influenced colorectal cancer differentiation but did not affect the predisposition to CRC. Moreover, this genetic association was detected exclusively in colon cancer but not in rectal cancer, indicating an anatomical site-specific effect of *CA9* gene polymorphisms on the progression of colorectal malignancies.

Through its catalytic and non-catalytic functions, CA9 is known to endow cancer cells with survival advantages in low-oxygen conditions and to confer a growing capability to disseminate 27. In addition, associations of *CA9* SNPs with different aspects of cancer development have been reported 15-20. Among these, rs12553173, a synonymous variant, was shown to correlate with improved median survival in patients with metastatic kidney cancer 19. Another non-coding variant located in the 3'-untranslated region of *CA9* gene, rs1048638, has been demonstrated to affect the susceptibility to liver 16 and cervical cancer 17. In the present study, we found that rs2071676 influenced colorectal cancer differentiation but did not affect the predisposition to CRC. This genetic variation (G>A) causes the substitution of valine by methionine at position 33 in the signal peptide of the protein product. Although the functional analysis of rs2071676 remain unavailable, this missense variation may disturb the removal of signal peptide after translocation of the nascent polypeptide into the endoplasmic reticulum (ER) lumen. Genetic alterations in either the signal peptidase recognition domain or hydrophobic region of signal peptides can impede cleavage of the signal peptide 28, 29. As a consequence, some protein products could be accumulated or degraded due to deficient glycosylation, inaccurate protein folding, and attenuated transport from ER to Golgi 30. In patients with retinitis pigmentosa, a pathogenic genetic variant that causes replacement of an arginine with a tryptophan in the signal sequence of the carbonic anhydrase 4 (*CA4*) gene resulted in a reduction of CA4 activity by virtue of a combination of decreased synthesis and accelerated turnover 31. Thus, we speculate that polymorphic genotype of rs2071676 may lead to accumulation of misfolded forms of CA9 in the ER of epithelial cells within the colon. Such chronic ER stress, in turn, contributes to altered adhesion and catalytic activity of CA9, ultimately affecting the progression of CRC. Determining the functional impact of rs2071676 on CA9 enzyme activity, the rates of biosynthesis, conversion of unfolded to mature enzyme, and turnover of CA9 will require further investigation.

Furthermore, it is intriguing that rs2071676 was exclusively associated with clinical variables of colon cancer but not with that of rectal cancer, revealing an anatomical site-specific effect of *CA9* gene polymorphisms on the progression of colorectal malignancies. Although both colon and rectal cancer develop in the large bowel and are often considered as a single tumor entity in all fields of basic and clinical research, substantial differences in molecular carcinogenesis exist 32. Compared to rectal cancer, colon cancer more commonly exhibited higher activity of MAPK signaling pathways 33, elevated expression of HOX gene family 34, and constitutively active forms of BRAF 35, 36 and KRAS 37. In addition to differential orchestration of many cancer hallmarks in tumors of the colon and rectum, these disturbed oncogenic signaling pathways also controlled the expression or stability of a major transcriptional inducer of CA9, hypoxia-inducible factor (HIF) 38-40, thereby leading to the fluctuations of local CA9 levels within CRC tumor microenvironment. These findings, in part, account for our observation that rs2071676 was associated with the progression of CRC in an anatomical site-specific manner.

This study unveiled a potential role of *CA9* gene polymorphisms in many aspects of colon cancer progression. Yet, extra efforts are required to address several limitations in the study. One is that the influence of *CA9* SNPs on CRC susceptibility might be underestimated due to the unavailability of data about the status of alcohol consumption and cigarette use for adjustment. Another caveat is that the mechanistic role of rs2071676 in tumor progression remains mostly unclear. Whether the substitution of valine by methionine due to the presence of polymorphic allele affects its expression, membrane translocation, or internalization needs to be further defined. Moreover, the results observed in this investigation might be not applicable to other racial groups except if replication studies are carried out.

In conclusion, our findings demonstrate an association of a *CA9* SNP, rs2071676, with cancer differentiation and invasiveness in tumors of the colon, highlighting an anatomical site-specific effect of *CA9* gene polymorphisms on the progression of colorectal malignancies.

## Figures and Tables

**Table 1:** The distributions of demographical and clinical characteristics in 470 controls and 470 patients with CRC

Variable	Controls (N=470), n (%)	Patients (N=470) (%)	p value
**Age (yrs)**			
<65	269 (57.2%)	249 (53.0%)	0.190
≥65	201 (42.8%)	221 (47.0%)	
**Gender**			
Male	292 (62.1%)	275 (58.5%)	0.257
Female	178 (37.9%)	195 (41.5%)	
**Tumor location**			
Rectum		107 (22.8%)	
Left colon		220 (46.8%)	
Right colon		143 (30.4%)	
**Stage**			
I+II		223 (47.4%)	
III+IV		247 (52.6%)	
**Tumor T status**			
T1-T2		113 (24.0%)	
T3-T4		357 (76.0%)	
**Lymph node status**			
N0		233 (49.6%)	
N1+N2		237 (50.4%)	
**Metastasis**			
M0		394 (83.8%)	
M1		76 (16.2%)	
**Lymphovascular invasion**			
No		258 (54.9%)	
Yes		212 (45.1%)	
**Perineural invasion**			
No		264 (56.2%)	
Yes		206 (43.8%)	
**Pathologic grading**			
Well		6 (1.3%)	
Moderately		428 (91.0%)	
Poorly		36 (7.7%)	

**Table 2:** Genotype Distributions of *CA9* Gene Polymorphisms in 470 Controls and 470 Patients with CRC

Variable	Controls (N=470), n (%)	Patients (N=470), n (%)	OR (95% CI)	AOR (95% CI)
**rs2071676**				
AA	130 (27.7%)	137 (29.1%)	1.000 (reference)	1.000 (reference)
AG	231 (49.1%)	218 (46.4%)	0.896 (0.661-1.212)	0.774 (0.472-1.269)
GG	109 (23.2%)	115 (24.5%)	1.001 (0.702-1.428)	0.770 (0.436-1.359)
AG+GG	340 (72.3%)	333 (70.9%)	0.929 (0.700-1.234)	0.773 (0.487-1.226)
**rs3829078**				
AA	438 (93.2%)	437 (93.0%)	1.000 (reference)	1.000 (reference)
AG	32 (6.8%)	33 (7.0%)	1.034 (0.624-1.711)	1.151 (0.504-2.629)
GG	0 (0%)	0 (0%)	---	---
AG+GG	32 (6.8%)	33 (7.0%)	1.034 (0.624-1.711)	1.151 (0.504-2.629)
**rs1048638**				
CC	411 (87.4%)	408 (86.8%)	1.000 (reference)	1.000 (reference)
CA	56 (11.9%)	57 (12.1%)	1.025 (0.692-1.520)	0.636 (0.302-1.314)
AA	3 (0.7%)	5 (1.1%)	1.679 (0.399-7.071)	1.219 (0.142-10.475)
CA+AA	59 (12.6%)	62 (13.2%)	1.059 (0.723-1.551)	0.680 (0.337-1.372)

The odds ratio (OR) with their 95% confidence intervals were estimated by logistic regression models. The adjusted odds ratio (AOR) with their 95% confidence intervals were estimated by multiple logistic regression models after controlling for age and gender.

**Table 3:** Distribution of the clinical status and CA9 rs2071676 genotype frequencies in 470 CRC patients

Variable	AA (N=137)	AG + GG (N=333)	OR (95% CI)	p value
**Stages**				
I+II	65 (47.4%)	158 (47.4%)	1.000 (reference)	p=1.000
III+IV	72 (52.6%)	175 (52.6%)	1.000 (0.671-1.326)	
**Tumor T status**				
T1+T2	28 (20.4%)	85 (25.5%)	1.000 (reference)	p=0.241
T3+T4	109 (79.6%)	248 (74.5%)	0.749 (0.462-1.215)	
**Lymph node status**				
Negative	69 (50.4%)	164 (49.2%)	1.000 (reference)	p=0.826
Positive	68 (49.6%)	169 (50.8%)	1.046 (0.702-1.557)	
**Metastasis**				
Negative	116 (84.7%)	278 (83.5%)	1.000 (reference)	p=0.751
Positive	21 (15.3%)	55 (16.5%)	1.093 (0.632-1.889)	
**Lymphovascular invasion**				
No	68 (49.6%)	190 (57.1%)	1.000 (reference)	p=0.142
Yes	69 (50.4%)	143 (42.9%)	0.742 (0.498-1.105)	
**Perineural invasion**				
No	72 (52.6%)	192 (57.7%)	1.000 (reference)	p=0.311
Yes	65 (47.4%)	141 (42.3%)	0.813 (0.545-1.213)	
**Cell differentiation**				
Well/Moderately	121 (88.3%)	313 (94.0%)	1.000 (reference)	**p=0.036**
Poorly	16 (11.7%)	20 (6.0%)	0.483 (0.242-0.963)	

**Table 4:** Distribution frequency of the clinical status and CA9 rs2071676 genotype frequencies in 470 CRC patients with different cancer site

Variable	Rectum (N=107)			Colon (N=363)		
	AA (N=31)	AG + GG (N=76)	p value	AA (N=106)	AG + GG (N=257)	p value
**Stages**						
I+II	20 (64.5%)	38 (50.0%)	p=0.172	45 (42.5%)	120 (46.7%)	p=0.461
III+IV	11 (35.5%)	38 (50.0%)		61 (57.5%)	137 (53.3%)	
**Tumor T status**						
T1+T2	11 (35.5%)	23 (30.3%)	p=0.599	17 (16.0%)	62 (24.1%)	p=0.090
T3+T4	20 (64.5%)	53 (69.7%)		89 (84.0%)	195 (75.9%)	
**Lymph node status**						
Negative	20 (64.5%)	40 (52.6%)	p=0.261	49 (46.2%)	124 (48.2%)	p=0.726
Positive	11 (35.5%)	36 (47.4%)		57 (53.8%)	133 (51.8%)	
**Metastasis**						
Negative	26 (83.9%)	63 (82.9%)	p=0.903	90 (84.9%)	215 (83.7%)	p=0.768
Positive	5 (16.1%)	13 (17.1%)		16 (15.1%)	42 (16.3%)	
**Lymphovascular invasion**						
No	23 (74.2%)	45 (59.2%)	p=0.144	45 (42.5%)	145 (56.4%)	**p=0.015^a^**
Yes	8 (25.8%)	31 (40.8%)		61 (57.5%)	112 (43.6%)	
**Perineural invasion**						
No	22 (71.0%)	48 (63.2%)	p=0.441	50 (47.2%)	144 (56.0%)	p=0.124
Yes	9 (29.0%)	28 (36.8%)		56 (52.8%)	113 (44.0%)	
**Cell differentiation**						
Well/Moderately	31 (100%)	75 (98.7%)	p=0.521	90 (84.9%)	238 (92.6%)	**p=0.024^b^**
Poorly	0 (0.0%)	1 (1.3%)		16 (15.1%)	19 (7.4%)	

^a^ OR (95% CI):0.570 (0.361-0.900); ^b^ OR (95% CI):0.449 (0.221-0.911).
